# Public Awareness and Utilization of 937-Telephone Health Services in the Kingdom of Saudi Arabia Before and During the COVID-19 Pandemic: Longitudinal Study

**DOI:** 10.2196/27618

**Published:** 2021-07-30

**Authors:** Saja A Al-Rayes, Arwa Alumran, Duaa Aljabri, Afnan Aljaffary, Ethar Aldoukhi, Zainab Alahmedalyousif, Reem Al Madani

**Affiliations:** 1 Health Information Management and Technology Department College of Public Health Imam Abdulrahman bin Faisal University Dammam Saudi Arabia; 2 Risk Management Unit, Directorate of Quality and Safety King Fahah Hospital of the University Imam Abdulrahman bin Faisal University Dammam Saudi Arabia

**Keywords:** awareness, COVID-19, Kingdom of Saudi Arabia, telehealth, telemedicine, utilization

## Abstract

**Background:**

Telehealth plays a key role in supporting health care systems and influencing methods of health care delivery. Government laws and medical operating protocols have been largely modified to provide remote care to reduce social contact and ensure a safer patient environment. In the Kingdom of Saudi Arabia (KSA), the Ministry of Health (MOH) introduced several forms of telemedicine as alternatives to face-to-face consultations in clinical settings.

**Objective:**

This study aimed to assess the awareness and utilization of telehealth services before and during the COVID-19 outbreak in the KSA.

**Methods:**

In this longitudinal study, we compared the awareness and utilization of 937-telephone health services (ie, a toll-free telephone service to provide medical and administrative health care services at any time for the population) before and during the COVID-19 outbreak in the KSA. Using a convenience sampling technique, a validated web-based questionnaire was distributed on social media platforms (Facebook, Twitter, and WhatsApp) at 2 timepoints: before (February 2019) and during (May 2020) the COVID-19 pandemic.

**Results:**

The study sample comprised a total of 1961 participants who completed the questionnaire before (n=1303, 66%) and during (n=658, 33%) the COVID-19 pandemic. Both awareness (before=46% vs during=78%) and utilization (before=42% vs during=48%) of the 937-telephone health services increased significantly during the pandemic (*P*<.001). No significant association of the awareness or utilization of 937-telephone health services before and during the COVID-19 pandemic was found with respect to the participants’ age, education level, having children, or having any chronic disease.

**Conclusions:**

Our findings indicate significant increases in the awareness and utilization of 937-telephone health services during the early days of the COVID-19 pandemic, suggesting an increase in public acceptance of the service and providing evidence of an equitable telemedicine service for the population. Further studies are needed to provide a deeper understanding of the barriers and facilitators to the use of 937-telephone health services for different groups of the population.

## Introduction

The COVID-19 pandemic has transformed the manner in which health care services are provided to patients worldwide. With the rapid spread of COVID-19 from 1 city (Wuhan, China) to almost all countries, governments worldwide implemented restrictive measures to reduce the contamination risk. Many countries adopted extreme prevention methods to prevent disease transmission, such as self-isolation and quarantine, social distancing, prioritizing health centers for COVID-19 cases, closing borders, and the complete lockdown of cities. In an attempt to reduce the opportunity of disease transmission, medical operating protocols have been largely modified to comply with control measures. Thus, the delivery of health care services has shifted toward the use of telemedicine and remote services for medical consultations [1-5].

Telemedicine was first described in the 1970s and was defined as the process of providing health care from a distance through the use of communications technology [6,7]. Telephone consultations can be considered a rudimentary form of telemedicine [8]. Telemedicine can be described as “the process whereby patients receive medical advice by one or more qualified healthcare professionals via the telephone” [9].

In the Kingdom of Saudi Arabia (KSA), the Ministry of Health (MOH) introduced several forms of telemedicine as alternatives to face-to-face consultations in clinical settings. More than 19 mobile apps have been released to provide web-based health services to smartphone users, who constitute 96% of the population [10]. Another key initiative to improve health care access for those without an internet connection was the establishment of 937—a free, confidential telephone service that provides medical and administrative health care services at any time. These 937-telephone health services are available 24/7 throughout the year and are provided in both Arabic and English. Calls are answered by health care professionals who can ask questions to assess the health problem and provide the caller advice on whether to handle the problem at home or to go to a hospital emergency room. The service provides four main subservices: (1) medical consultation, (2) reporting complaints about public and private health facilities, (3) appointment requests for primary health centers and smoking cessation clinics, and (4) responding to inquiries such as requesting information about infectious diseases, toxins, and medicines and providing technical support for MOH mobile apps.

The 937-telephone health services are not limited to the telephone number “937” but are rather linked with a mobile app with an instant messaging feature, which allows patients to send photographs or videos during medical consultations (eg, of a skin rash). The service is also accessible for deaf people and those with hearing impairment through a special mobile app “Ishara” (ie, “sign”), through which a service provider launches a video call to connect the patient to the 937-telephone health care services and provides a sign-language translation.

In the months before the pandemic, the 937-telephone health services were not in effective use in the KSA, with only approximately 46% of the population reporting awareness of the service and only 20% using it [11]. Given that the first part of this study was conducted before the COVID-19 outbreak, we were able to assess whether awareness and utilization of the 937-telephone service had increased during the pandemic. This study aimed to assess the awareness and utilization of the 937-telephone health services before and during the COVID-19 outbreak in the KSA, and we hypothesized that awareness and utilization of the 937-telephone health services increased during the pandemic.

## Methods

### Study Design

This study used a longitudinal approach, where data were collected at two timepoints: before (February 2019) and during the COVID-19 pandemic (May 2020).

### Participants

The survey included 2 cohorts of citizens and residents of the KSA. Participants were selected using a nonprobability sampling technique, specifically convenience sampling.

### Variables

The questionnaire measured awareness and utilization of the 937-telephone health services in the KSA. Awareness was measured by asking, “Are you aware of the Ministry of Health 937-telephone health service (yes vs no)?” Utilization was measured by asking, “Have you ever contacted 937-telephone health services (yes vs no)?” Participants who answered “yes” to the second question were asked a follow-up question regarding the purpose of their call (eg, for a medical consultation, complaints, appointments, or a general inquiry).

In addition, data on independent variables were collected to assess their association with awareness and utilization. These included data on age (age groups: ≤19 years, 20-29 years, 30-39 years, 40-49 years, 50-59 years, and ≥60 years), gender (male or female), level of education (at least high school, diploma, bachelor’s degree, or graduate degree), area of residence (Eastern, Western, Northern, Southern, and Central regions), nationality (Saudi citizen or resident), having children younger than 18 years old (yes vs no), and previous diagnoses of chronic disease (yes vs no). 

### Data Source or Instrument

As digital communication channels have been increasingly used to recruit study participants in recent studies, especially during the COVID-19 pandemic, we used a web-based questionnaire for data collection. Three methods were used to validate the survey: face validity (to test the clarity and relevancy of the content and the scale overall layout), content validity, and linguistic validity. The validation process has been previously described by Alrayes et al [11].

### Sample Size

The questionnaire was distributed on social media platforms (ie, Facebook, Twitter, and WhatsApp) with no inclusion or exclusion criteria; hence, formal sample size calculations were not feasible. As an alternative, we followed a previously recommended guideline [12] for sample sizes: 100=“fair,” 200=“good,” 500=“very good,” and >1000=“excellent.” 

### Statistical Analysis

The data were analyzed using SPSS (IBM Corp) [13]. Demographic characteristics, awareness, and utilization were analyzed using descriptive analysis. Chi-square tests were used to assess the difference in awareness and utilization of the 937-telephone health services before and during the pandemic. In addition, factors influencing the awareness and utilization of the 937-telephone health services were assessed using chi-square tests, and *P* values less than .05 were considered significant for all analyses.

### Ethical Considerations

All participants were asked for their voluntary participation and consent before filling out the questionnaire and were free to terminate the survey at any time. Ethics approval was obtained from the institutional review board of Imam Abdulrahman Bin Faisal University (protocol# IRB-UGS-2018-03-294).

## Results

### Participants

The study sample included 1961 participants who completed the questionnaire before (n=1303, 66%) and during (n=658, 34%) the COVID-19 pandemic. Nonsignificant differences were observed between the population cohorts, which indicated that the study samples were homogenous.

### Descriptive Data

Most study participants were women (n=1488, 76%), and approximately one-third were 20-29 years old (n=628, 32%). Half of the participants indicated that they had at least 1 child (n=1008, 51%), and most did not have a chronic disease (n=1705, 87%). The majority of study participants resided in the Eastern region (n=1624, 83%) (Table 1).

**Table 1:** Awareness of 937-telephone health services in the Kingdom of Saudi Arabia before and during COVID-19 pandemic.

Variables		Overall (n=1961), n (%)	Aware (n=1113), n (%)	Awareness of the 937-telephone health service (n=1113)		Chi-square (*df*)		*P* value	
				Before the COVID-19 pandemic (n=601), n (%)	During the COVID-19 pandemic (n=512), n (%)				
**Gender**							11.5 (1)		.001^a^
	Female	1488 (76)	813 (73)	464 (77)	349 (68)				
	Male	473 (24)	300 (27)	137 (23)	163 (32)				
**Age (years)**							5.2 (5)		.39
	≤19	117 (6)	40 (4)	18 (3)	22 (4)				
	20-29	628 (32)	332 (30)	187 (31)	145 (28)				
	30-39	454 (23)	300 (27)	168 (28)	132 (26)				
	40-49	443 (23)	258 (23)	136 (23)	122 (24)				
	50-59	262 (13)	153 (14)	80 (13)	73 (14)				
	≥60	57 (3)	30 (3)	12 (2)	18 (4)				
**Having children**									
	Yes	1008 (51)	632 (57)	352 (59)	280 (55)	1.7 (1)		.19	
	No	953 (49)	481 (43)	249 (41)	232 (45)				
**Nationality**									
	Saudi citizen	1907 (97)	1090 (98)	594 (99)	496 (97)	5.3 (1)		.02^a^	
	Resident	54 (3)	23 (2)	7 (1)	16 (3)				
**Area of residence**									
	East	1624 (83)	916 (82)	462 (77)	454 (89)	45.4 (3)		<.001^a^	
	Central	170 (9)	113 (10)	94 (16)	19 (4)				
	West	112 (5)	54 (5)	32 (5)	22 (4)				
	North and south	55 (3)	30 (3)	13 (2)	17 (3)				
**Education level**							3.4 (3)		.33
	High school or lower	399 (20)	199 (18)	97 (16)	102 (20)				
	Diploma	225 (12)	136 (12)	75 (13)	61 (12)				
	Bachelor’s degree	1,154 (59)	641 (58)	349 (58)	292 (57)				
	Graduate degree	183 (9)	137 (12)	80 (13)	57 (11)				
**Diagnosis of a chronic disease**							1.0 (1)		.33
	Yes	256 (13)	153 (14)	77 (13)	76 (15)				
	No	1705 (87)	960 (86)	524 (87)	436 (85)				

^a^Significant differences.

### Awareness and Utilization of the 937-Telephone Service

Among the participants who responded during the pandemic, 512 (78%) indicated that they were aware of the 937-telephone health services, while only 46% of those who responded before the pandemic were aware of the service, which indicates a significant increase in awareness since the pandemic emerged in the KSA (*χ*^2^_1_=178.9; *P*<.001) (Table 2). Awareness of the 937-telephone health services was higher among women (68%), those aged 20-29 years (28%), and those with at least 1 child (55%) (Table 1).

**Table 2:** Influence of the COVID-19 pandemic on the awareness and utilization of the 937-telephone health services in the Kingdom of Saudi Arabia.

Variables		Overall, n (%)	Before the COVID-19 pandemic, n (%)	During the COVID-19 pandemic*,* n (%)	Chi-square (*df*)		*P* value	
**Aware of the 937-telephone health services**						178.9 (1)		<.001^a^
	Yes	1113 (57)	601 (46)	512 (78)				
	No	848 (43)	702 (53)	146 (22)				
**Using the 937-telephone health services**						76.6 (1)		<.001^a^
	Yes	509 (26)	258 (42)	251(48)				
	No	1452 (74)	1045 (58)	407 (52)				
**Reasons for using the 937-telephone health services**								
	Consultations	430 (84)	218 (84)	212 (84)	0.00 (1)		.99	
	Complaints	307 (60)	123 (48)	184 (73)	34.9 (1)		<.001^a^	
	Appointments	234 (46)	92 (36)	142 (57)	22.4 (1)		<.001^a^	
	Inquiries	305 (60)	137 (53)	168 (67)	10.1 (1)		<.001^a^	

^a^Significant differences.

Although the percentage of respondents utilizing the 937-telephone health services was similar before and during the pandemic (42% and 48%, respectively), this difference was still significant (*χ*^2^_1_=76.6; *P*<.001). Utilization during the pandemic was higher among women (n=146, 58%), those aged 30-39 years (n=85, 34%), and those with at least 1 child (n=161, 64%) (Table 3).

**Table 3:** Utilization of 937-telephone health services in the Kingdom of Saudi Arabia before and after COVID-19 pandemic.

Variables		Overall utilization (n=509), n (%)	Utilization of the 937-telephone health service		Chi-square (*df*)	*P* value
			Before the COVID-19 pandemic (n=258), n (%)	During the COVID-19 pandemic, (n=251), n (%)		
**Gender**					30.3 (1)	<.001^a^
	Female	354 (70)	208 (81)	146 (58)		
	Male	155 (30)	50 (19)	105 (42)		
**Age (years)**					4.4 (5)	.49
	≤19	12 (2)	7 (3)	5 (2)		
	20-29	125 (25)	68 (26)	57 (23)		
	30-39	178 (35)	93 (36)	85 (34)		
	40-49	119 (23)	59 (23)	60 (24)		
	50-59	65 (13)	28 (11)	37 (15)		
	≥60	10 (2)	3 (1)	7 (3)		
**Having children**					0.4 (1)	.55
	Yes	333 (65)	172 (67)	161 (64)		
	No	176 (35)	86 (33)	90 (36)		
**Nationality**					1.9 (1)	.17
	Saudi citizen	504 (99)	257 (99)	247 (98)		
	Resident	5 (1)	1 (1)	4 (2)		
**Area of residence**					26.6 (3)	<.001^a^
	East	416 (82)	196 (76)	220 (88)		
	Central	63 (12)	50 (19)	13 (5)		
	West	20 (4)	10 (4)	10 (4)		
	North and south	10 (2)	2 (1)	8 (3)		
**Education level**					0.9 (3)	.83
	High school or lower	90 (18)	47 (18)	43 (17)		
	Diploma	64 (13)	31 (12)	33 (13)		
	Bachelor’s degree	288 (57)	143 (55)	145 (58)		
	Graduate degree	67 (13)	37 (14)	30 (12)		
**Diagnosis of a chronic disease**					0.3 (1)	.61
	Yes	73 (14)	35 (14)	38 (15)		
	No	436 (86)	223 (86)	213 (86)		

^a^Significant differences.

### Reasons for Using the 937-Telephone Health Service

When assessing the reasons for using the 937-telephone health services, 4 service areas were considered: consultations, complaints, appointments, and inquiries (Table 2). Use of the service for complaints (*χ*^2^_1_=35.7; *P*<.001), appointments (*χ*^2^_1_=20.7; *P*<.001), and inquiry (*χ*^2^_1_=38.6; *P*<.001) was significantly different before and during the pandemic, while there was no significant difference in the use of the service for consultations before and during the pandemic (*χ*^2^_1_=3.4; *P*=.07).

As shown in Table 2, use of the service for complaints increased from 45% before to 63% during the COVID-19 pandemic. In addition, use of the service to schedule appointments increased from 27% before to 39% during the COVID-19 pandemic. Finally, participants’ utilization of the service to make inquiries increased from 48% before to 67% during the COVID-19 pandemic.

### Factors Influencing the Awareness or Utilization of the 937-Telephone Health Service

In bivariate analysis, several variables significantly influenced participants’ awareness before and during the COVID-19 pandemic. These variables were the following: gender, nationality, and area of residence. Women were generally more aware about the 937-telephone health services than men, regardless of the time point in the study (*χ*^2^_1_=11.5; *P*=.001).

Area of residence was significantly associated with awareness of the 937-telephone health services (*χ*^2^_3_=45.4; *P*<.001). There was a notable increase in awareness across all areas in the KSA after the COVID-19 pandemic emerged (Table 1). Awareness in the Eastern region increased from 77% before to 89% during the COVID-19 pandemic.

Two factors were significantly associated with the utilization of the 937-telephone health services in this study: gender (*χ*^2^_1_=30.3; *P*<.001) and area of residence (*χ*^2^_3_=26.6; *P*<.001). Female participants utilized the service more than male participants before and during the COVID-19 pandemic; however, the difference was more significant before the pandemic (81% vs 19% and 58% vs 42%, respectively). Utilization of the service increased among men during the COVID-19 pandemic. In addition, utilization of the 937-telephone health services increased significantly in the Eastern region from 76% before to 88% during the COVID-19 pandemic (Table 3).

No other variables assessed in the study showed a significant association with the awareness or utilization of the 937-telephone health services before or during the COVID-19 pandemic. These variables include age, having children, level of education, and previous diagnoses of chronic disease. This suggests relatively equal awareness and utilization of the service across the population.

## Discussion

### Principal Findings

The 937-telephone health service has thrived during the COVID-19 pandemic owing to its ability to deliver remote health care services to all patients in the community. Public administrations in the United States, United Kingdom, and Australia are investing in telemedicine to reduce the volume of patients visiting emergency departments for nonurgent concerns and to limit virus transmission [14]. This study aimed to compare the awareness and utilization of the 937-telephone health services before and during the COVID-19 pandemic in the KSA.

Since the COVID-19 outbreak in the KSA, the government has employed a number of policies to implement some level of lockdown, including the cancellation of regular outpatient visits, the prioritization of hospital usage for COVID-19 cases, and the expansion and improvement of available telehealth services. Several awareness programs were initiated by the MOH for public education and COVID-19 prevention, aiming to distribute proper information and increase future preparedness. One such MOH initiative was a public awareness campaign encouraging the public to use the existing 937-telephone health services for any inquiry regarding COVID-19, such as symptoms, methods of prevention, and the required course of action on the occurrence of any of its symptoms. Our study findings showed a significant increase in both awareness and utilization levels before and during the pandemic (46% vs 78% awareness and 42% vs 48% utilization). This might have resulted from the increased awareness campaigns by the government to encourage use the 937-telephone health service as an alternative approach for nonemergency concerns.

A parallel increase in the levels of awareness of the role of telehealth in relation to COVID-19 was noted in Australia [3]. Furthermore, 59% of surveyed individuals reported being aware of the 111-telephone health services of the National Health Service in the United Kingdom and 9% reported having used them [15]. Public perception of telephone consultations has been generally positive [16]. Many people value the opportunity to obtain expert advice over the telephone as an initial response to the development of new symptoms. Cited benefits include reduced waiting and travel times, convenience, and flexibility [17,18].

Our findings show that the population engaged with the 937-telephone health services differently during the pandemic. The telephone service was mostly used for consultations (84%), followed by complaints (73%), inquiries (67%), and appointment requests (57%). While utilization of the service for consultations was the same before and during the COVID-19 pandemic, its utilization for other reasons increased significantly during the pandemic. This was expected, as during the early days of the pandemic, the population might have been uncertain, owing to inconsistent, unverified, and conflicting information from various sources [19]. Thus, the ability to obtain information from an accurate source such as the MOH telephone service might have helped overcome public uncertainty.

We found that gender played a role in the awareness of 937-telephone health services, with women being more aware than men. This finding aligns with that of another study that reported that women are more likely to engage in eHealth than men [20]. In contrast, in 2017, Khatun et al [21] found that men were more aware of existing health care services, and Jung et al [22] did not find significant differences in telemedicine awareness based on gender.

Another interesting finding, although not significant, was that awareness and utilization of the 937-telephone health services were lower among older adults. Although using the 937-telephone health services does not require an internet connection or computer skills, lesser access was noted among older people. This finding is concurrent with that of Knowles et al [15] who reported that older people were less likely to use telephone-accessed health care (than younger people) and preferred face-to-face contact. Jung et al [22] reported that 77% of younger participants (aged <50 years) preferred to use telemedicine compared to 62.5% of older participants (those aged ≥50 years). Furthermore, El-Mahalli et al [23] reported that those between 30 and 50 years of age were most likely to use telemedicine. Cultural factors might have contributed to this finding as younger adults often take care of seniors, making it unnecessary for them to use the service [23]. In addition, as older people are slower in adopting new technologies than younger adults, the web-based questionnaire might not have reached the intended older population, thus causing a nonresponse bias [24]. However, as the population ages and the prevalence of long-term conditions increases, telehealth is a particularly valuable tool that could help elderly people maintain their health and benefit those with limited mobility [25]. Thus, understanding barriers and facilitators for telehealth use among older individuals may be important.

Another interesting finding was that among study participants, area of residence was associated with the awareness of the 937-telephone health services. Those residing in the Eastern region of the KSA knew more about the service than those residing in other regions. Jung et al [22] reported that awareness of telehealth services is affected by where a person lives; for example, large cities compared to rural areas. This is most likely due to financial support in large cities [3]. In contrast, another study reported that remote regions were more aware of telehealth services [22].

Our study confirms the increase in awareness and utilization of telehealth, driven by the emergence of the COVID-19 pandemic, which has transformed health care delivery. To our knowledge, this study is the first in the KSA to assess awareness and utilization of 937-telephone health services during the COVID-19 pandemic and to compare current levels of awareness and utilization with those before the pandemic. In addition, the sample size included in the study is large (n=1961), which provides wide representation of the population.

### Implications for Telephone Health Care Services During Pandemics

With the increased demand for emergency care services and government enforcement of extreme physical distancing measures, health care providers are seeking alternative strategies for providing cost-effective and safe care. Policymakers are implementing telephone consultations as a substitution for face-to-face consultations, with a goal of maintaining continuity of care in a less expensive and safer environment. Currently, the telephone service provides nonurgent care; however, the onset of the COVID-19 pandemic has accelerated the telehealth revolution and rendered the consideration of innovative models of care essential. Reliable and valid screening instruments for telephone use are required to allow health care providers to make comprehensive assessments during the remote visit. Hence, the use of web-based apps to expand telehealth use and build its capacity within the health care delivery system is important.

### Limitations

However, there are several limitations of note. First, our study population consisted predominantly of women (76%), which may have biased our results; in particular, those related to differences in awareness by gender. However, studies have shown that women tend to be more proactive regarding their health than men [20]. Second, ethnicity might have a potential role in influencing the utilization of telephone services; however, because ethnicity categorization is not used in the official censuses in the KSA and thus might be culturally unacceptable, it was not be feasible to examine this issue in this study. Instead, a more culturally acceptable alternative, nationality, was considered when constructing the study questionnaire as an indirect reflection of a participant’s ethnicity. In addition, area of residence is also captured and is considered a cultural categorization in the KSA. Third, the use of the convenience sampling technique, which yielded a sample in which 83% of the participants lived in the Eastern region, likely contributed to the oversampling of participants in this region, thus limiting our inference on differences observed by area of residence and awareness of the 937-telephone health services. Fourth, data on utilization and awareness during the COVID-19 pandemic were collected in March 2020. The first local COVID-19 case was reported on March 2, 2020, and shortly thereafter, the World Health Organization declared COVID-19 a pandemic (March 11, 2020) [26]. It is possible that awareness and utilization of 937-telephone health services increased over the following months. Finally, it was difficult to calculate the response rate as the questionnaire was distributed on the internet, and no power calculation for the sample size estimation was undertaken. However, the sample size fell within the normative ranges deemed “excellent” (>1000) for common research designs [12,27].

### Conclusions

This study found significant increases in the awareness and utilization of 937-telephone health services in the KSA, with nonsignificant differences across population groups before and during the COVID-19 pandemic. This suggests an increase in public acceptance of the service and offers evidence for the availability of an equitable telephone-accessed health service for the population. In addition, the utilization of 937-telephone health services for medical appointments and inquiries increased significantly during rather than before the COVID-19 pandemic. Further studies are needed to provide a deeper understanding of the barriers and facilitators for the utilization of the 937-telephone health services among different population groups.

## Abbreviations

KSA: Kingdom of Saudi Arabia
MOH: Ministry of Health
